# Gene aberrations of *RRM1* and *RRM2B* and outcome of advanced breast cancer after treatment with docetaxel with or without gemcitabine

**DOI:** 10.1186/1471-2407-13-541

**Published:** 2013-11-12

**Authors:** Charlotte LT Jørgensen, Bent Ejlertsen, Karsten D Bjerre, Eva Balslev, Dorte L Nielsen, Kirsten V Nielsen

**Affiliations:** 1 Department of Pathology, Herlev University Hospital, Copenhagen, Denmark; 2 Danish Breast Cancer Cooperative Group (DBCG) Registry, Rigshospitalet, Copenhagen, Denmark; 3 Department of Oncology, Herlev University Hospital, Copenhagen, Denmark; 4 VIF, Centre for Innovation and Research, Capital Region of Denmark, Copenhagen, Denmark

**Keywords:** Docetaxel, FISH, Gemcitabine, Gene aberrations, Metastatic breast cancer, Ribonucleotide reductase, *RRM1*, *RRM2B*

## Abstract

**Background:**

The purpose of the present study was to retrospectively evaluate whether copy number changes of the genes encoding the ribonucleotide reductase subunit M1 (*RRM1*) and/or subunit M2B (*RRM2B*) predict sensitivity to gemcitabine administered in combination with docetaxel compared to single agent docetaxel in advanced breast cancer patients.

**Methods:**

Primary tumor samples from patients randomly assigned to gemcitabine plus docetaxel or docetaxel alone were analyzed for *RRM1* and *RRM2B* copy number changes using Fluorescence *In Situ* Hybridization (FISH) technology with probes covering respectively *RRM1* at 11p15.5 and a reference probe covering the centromere of chromosome 11 (CEN-11), and *RRM2B* at 8q22.3 and a reference probe covering the centromere of chromosome 8 (CEN-8). The assays were validated in a material of 60 normal breast samples. Time to progression (TTP) was the primary endpoint. Overall survival (OS) and response rate (RR) were secondary endpoints. Associations between *RRM1/*CEN-11 and/or *RRM2B*/CEN-8 ratios and time-to-event endpoints were analyzed by unadjusted and adjusted Cox proportional hazards regression models. Heterogeneity of treatment effects on TTP and OS according to gene status were investigated by subgroup analyses, and the Wald test was applied. All statistical tests were two-sided.

**Results:**

FISH analysis for both *RRM1* and *RRM2B* was successful in 251 patients. *RRM1* and *RRM2B* aberrations (deletions and amplifications) were observed in 15.9% and 13.6% of patients, respectively. *RRM1* aberrations were associated with a decreased OS in the time interval 1.5-7.4 years (hazard ratio = 1.72, 95% confidence interval = 1.05-2.79, P = 0.03). *RRM2B* aberrations alone or in combination with *RRM1* aberrations had no prognostic impact in terms of TTP or OS. RR was not different by gene status*.* No significant differences were detected in TTP or OS within subgroups according to gene status and chemotherapy regimen.

**Conclusions:**

This study demonstrated the presence of *RRM1* and *RRM2B* copy number changes in primary breast tumor specimens. Nevertheless, we found no support of the hypothesis that aberrations of *RRM1* or *RRM2B*, neither individually nor in combination, are associated with an altered clinical outcome following chemotherapy with gemcitabine in combination with docetaxel compared to docetaxel alone in advanced breast cancer patients.

## Background

Ribonucleotide reductase (RNR) catalyzes the formation of deoxyribonucleotides, thus is a key enzyme during DNA synthesis and repair [1,2], and is the specific cellular target of gemcitabine [3]. Mammalian cells enclose three non-identical subunits of RNR: one homodimeric large subunit (RRM1) carrying the catalytic site, and two variants of a homodimeric small subunit (RRM2 and RRM2B) containing a tyrosyl free radical essential for catalysis [1,2]. As a nucleoside analogue, gemcitabine is intracellularly phosphorylated into the active metabolite gemcitabine diphosphate, which is recognized by RNR as a normal substrate and reacts with the substrate-binding catalytic site of the RRM1 subunit thereby inactivating the enzyme [3-5]. Preclinical research has indicated that increased tumor expression of RRM1 is the major determinant of resistance to gemcitabine [6], and two cell line studies have demonstrated an association between gemcitabine resistance and gene amplification of *RRM1*[7,8]. In addition, low/negative RRM1 mRNA and protein levels have been reported to correlate significantly with higher response rate and a better prognosis in lung cancer patients treated with gemcitabine-based chemotherapy, and in pancreatic and biliary cancer patients treated with gemcitabine alone [6,9]. However, there are reports, including a prospectively conducted phase III clinical trial, of RRM1 either not being significantly associated or possibly oppositely associated with survival in lung cancer patients receiving a gemcitabine-containing regimen [10-13]. A possible association between mRNA and protein expression of RRM2 or the RRM2B subunit and the effect of gemcitabine is less well studied and has not been addressed in properly sized randomized trials and results from pilot trials are inconsistent [10,14-18].

Thus, an unambiguous association has not been established between mRNA and protein expression of RNR and benefit from gemcitabine. The underlying copy number changes of the genes encoding the subunits of RNR may be determined more reproducible. To address this issue, gene copy number alterations of the enzyme subunits as determined by Fluorescence *In Situ* Hybridization (FISH) technology were evaluated in archival primary tumor samples from patients with locally advanced and metastatic breast cancer randomized to docetaxel alone (D) versus gemcitabine plus docetaxel (GD) [19]. Secondarily, the overall prognostic impact of RNR gene copy number variation in these patients receiving chemotherapy was investigated. Furthermore, as chromosomal instability and copy number alterations have previously been reported to be more prone to accumulate in highly proliferative subtypes (i.e. non-luminal A) [20,21], we analyzed the genomic landscape of RNR gene copy number changes in relation to PAM50 classified intrinsic subtypes (Jørgensen et al. manuscript provisionally accepted for publication) to investigate subtype specific patterns.

## Methods

### The patient study cohort

The Danish Breast Cancer Cooperative Group (DBCG) phase III multicenter 0102 trial randomized 337 women with advanced breast cancer to D versus GD. The trial has been described in detail previously [19]. Patients were randomly assigned to D (100 mg/m^2^) day 1, or G (1000 mg/m^2^) days 1 and 8 plus D (75 mg/m^2^) day 8, every 3 weeks. Prior chemotherapy with a non-taxane regimen was allowed either adjuvant or as first-line. The study was conducted in accordance with the Declaration of Helsinki, and all patients gave their signed informed consent prior to study entry. DBCG prepared the original protocol as well as the biomarker supplement, and the Danish National Committee on Biomedical Research Ethics approved the original protocol in addition to the add-on (KF-02-045-01 and KF-12-315632/H-KF-02-045-01) before activation.

### Specimen collection

Formalin-fixed, paraffin-embedded (FFPE) primary tumor blocks from participating patients were retrospectively collected from the archives of pathology departments throughout hospitals of Denmark. All samples were analyzed as tissue microarrays (TMAs), except six samples analyzed as whole sections because of small tumor size. Areas from the periphery of invasive tumor in donor blocks were identified on haematoxylin-eosin stained sections and TMAs were designed in the same manner as described previously [22]. From available and suitable blocks, 2.0 mm core TMAs were constructed by means of a TMA builder (Beecher Instrument ATA-27) by a histopathology skilled biologist (CLTJ) under supervision of a pathologist (EB). Consecutive 3 μm sections from each TMA block and the six whole tissue blocks were cut for processing of FISH.

### Assay validation cohort

For assay validation we analyzed the RNR candidate gene copy number variation in 60 TMA samples of normal breast gland tissue as previously described in Nielsen et al. 2011 [23].

### Pilot study

A pilot study was conducted to evaluate the existence of copy number changes of the genes encoding the subunits of RNR (*RRM1, RRM2, RRM2B*) as determined by FISH in primary tumor samples from 49 of the 337 advanced breast cancer patients treated with D versus GD [19]. Genetic aberrations were observed regarding *RRM1* and *RRM2B* (data not shown). Despite the fact that a relationship between the expression of the RRM2 subunit and gemcitabine has also been suggested, no copy number changes were found regarding *RRM2* during the pilot study (data not shown), and further evaluation of the entire cohort concerned *RRM1* and *RRM2B* copy number changes only.

### *RRM1* and *RRM2B* FISH assay development

The FISH assays were developed based on bacterial artificial chromosome (BAC) DNA fluorescent probes covering the sequence of the *RRM1* gene at 11p15.5 (BAC clones RP11-23F23 and RP11-982G19) and the *RRM2B* gene at 8q22.3 (BAC clone RP11-318G5), respectively. As reference, centromeric Peptide Nucleic Acid (PNA) probes specific for chromosome 11 (CEN-11) and 8 (CEN-8) were applied. The *RRM1* and *RRM2B* targeted BAC clones were labeled with Texas Red fluorochrome by Nick translation. Centromere targeted reference probes were based on a mixture of PNA oligoes labeled with fluorescein isothiocyanate. As blocking agent a mixture of alu PNA oligoes was used.

### FISH analysis

FISH analysis was performed on samples from tumor tissue and normal tissue using Dako Histology FISH accessory kit (K5599, Dako A/S, Glostrup, Denmark) according to the manufacturer’s instructions and as described previously [23]. Evaluation of slides was done according to the topoisomerase II-alpha (*TOP2A*) FISH scoring guidelines (from Dako K5333 USA package insert, 1^st^ edition 2008.01.18). Sufficient nuclei were included until a total of 60 red gene probe signals were counted along with the green reference probe signals in the corresponding nuclei. The ratio was calculated as the number of signals for the gene probe divided by the number of signals for the centromere reference probe. A case was considered to be amplified if the gene/centromere ratio was 2:1 or greater and deleted if the ratio was below 0.8:1, and otherwise normal. For explorative analysis, gene amplification was defined by a ratio of 1.5:1 or greater and gene deletion by a ratio below 0.9:1.

### Statistical analysis

For statistical analysis, data were dichotomized into overall genetic changes (amplification and deletion) and no genetic changes of *RRM1* or *RRM2B,* respectively. Combined genetic status of *RRM1* and *RRM2B* was dichotomized as 2R aberrant (amplification and deletion of one or both genes) and otherwise as 2R normal (normal copy number status of both genes).

Associations between gene status and prognostic and demographic variables of the main study [19] including PAM50 intrinsic subtype (Jørgensen et al. manuscript provisionally accepted for publication) were assessed. Associations between gene status and categorical variables (regimen, hormone receptor status, human epidermal growth factor receptor 2 (*HER2*) status, type of metastatic site, stage of disease, previous chemo-, hormonal-, and radio-therapy, and PAM50 intrinsic subtype) were evaluated by Fisher’s exact test, whereas associations between gene status and ordinal and interval variables (ECOG performance status, age at randomization, number of metastatic sites, and disease-free interval) were evaluated by the Wilcoxon rank-sum test.

Time to progression (TTP) was the primary endpoint for the original trial as well as for this biomarker sub-study [19], and overall survival (OS) and response rate (RR) were secondary endpoints. TTP was measured from random assignment to date of documented progression with censoring at date of last visit or at date of death. OS was calculated from date of random assignment to date of death with censoring for surviving patients at last visit date. Time-to-event endpoints (TTP and OS) were estimated by the Kaplan-Meier method, and association with gene status was assessed by the log-rank test. Analyses of gene status were done unadjusted and adjusted for preselected covariates in multivariate Cox proportional hazards models. The preselected covariates were those found to be significant in the previous analysis of the main study [19] including treatment regimen, disease type (visceral vs nonvisceral), stage of disease, performance status, and number of metastatic sites, in addition to PAM50 intrinsic subtype (Jørgensen et al. manuscript provisionally accepted for publication). The adjusted model was further stratified for previous chemotherapy [19]. The assumption of proportional hazards was assessed by Schoenfeld residuals. Subgroup analyses were done to assess whether treatment effects on TTP and OS varied according to gene status or the levels of preselected covariates. The multivariate Cox proportional hazards model was extended by one interaction term at a time and the interaction terms were tested using the Wald test.

RR was evaluated for patients with measurable disease. The overall RR was defined as a complete or partial response according to RECIST criteria, version 1.0. RRs were compared by using Fisher’s exact test.

Statistical analyses were conducted using the SAS version 9.2 software package (SAS Institute, Cary, NC, USA). All statistical tests were two sided, and P <0.05 considered statistically significant. Reporting Recommendations for Tumor Marker Prognostic Studies (REMARK) were adhered where applicable [24]. The design of the study is prospective-retrospective as described in Simon et al. [25].

## Results

**Figure 1:**
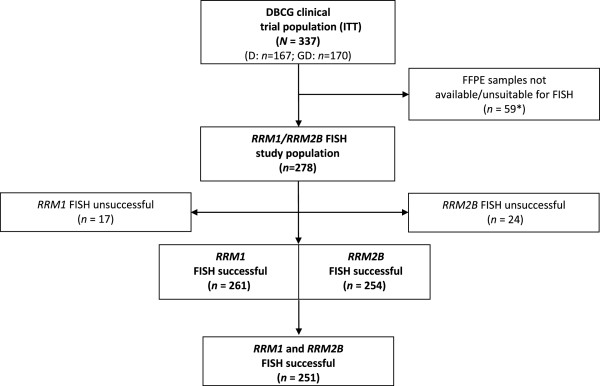
**Flow diagram of the presented study.** *Patients were withdrawn for one of the following reasons: archival tissue not available (n = 36), no tumor cells in available samples (n = 8), only needle biopsies available (n = 12), tissue samples available from metastasis only (n = 3). Abbreviation: D, docetaxel; DBCG, Danish Breast Cancer Cooperative Group; FFPE, formalin-fixed, paraffin-embedded; GD, gemcitabine plus docetaxel; FISH, fluorescence *in situ* hybridization; *RRM1*, ribonucleotide reductase M1 subunit; *RRM2B*, ribonucleotide reductase M2B subunit.

For the present study FFPE primary tumor tissue was available from 278 (82%) of the 337 patients enrolled in the intention to treat population (Figure 1). FISH analysis for *RRM1* and *RRM2B* was successful in 261 (94%) and 254 (91%) of the 278 patients, respectively. 251/278 patients (90%) had measurements of both genes and were included in the prespecified prospective-retrospective analysis.

Tumor tissue from patients who relapsed after primary mastectomy or breast conserving surgery was available from 229 (76%) compared to 22 (65%) from patients with locally advanced disease at diagnosis. As a consequence the 251 FISH assessable patients differed from the 86 non-assessable patients (P < 0.05) with regard to prior (neo)adjuvant chemotherapy, adjuvant hormonal therapy, and adjuvant radiotherapy, but not for other assessed parameters (Additional file 1: Table S1).

**Figure 2:**
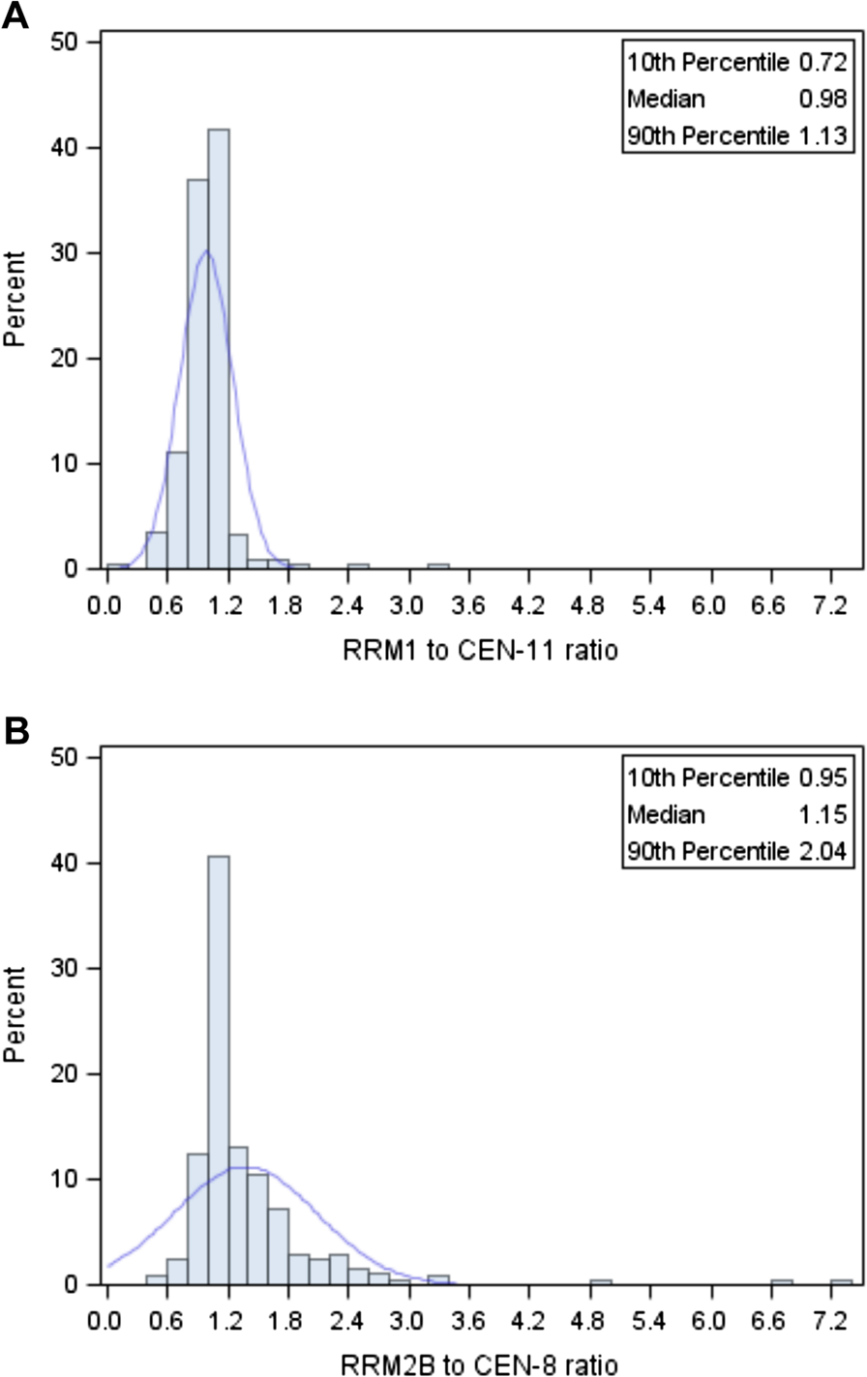
**Distribution of FISH ratios in the 251 FISH assessable primary breast tumor samples. (A)** Distribution of *RRM1*/CEN-11 ratios and **(B)** distribution of *RRM2B*/CEN-8 ratios. Abbreviation: CEN-8, centromere of chromosome 8; CEN-11, centromere of chromosome 11; FISH, fluorescence *in situ* hybridization; *RRM1*, ribonucleotide reductase M1 subunit; *RRM2B*, ribonucleotide reductase M2B subunit.

**Figure 3:**
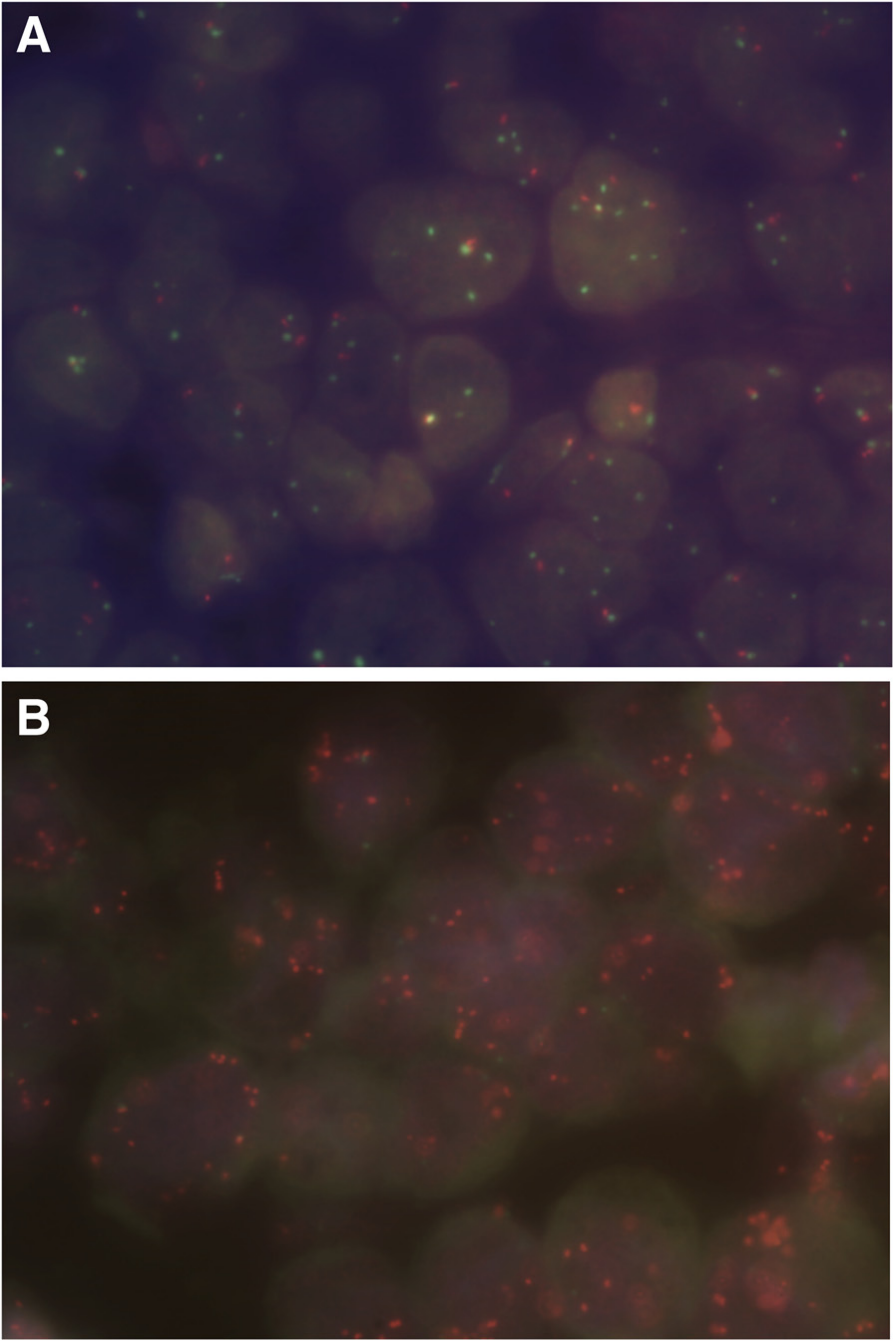
**Photomicrographs demonstrating examples of *RRM1 *and *RRM2B *status in invasive breast tumors.** Samples were analyzed by FISH using Texas Red (*RRM1* or *RRM2B*) and fluorescein isothiocyanate (chromosome 11and 8) labeled probes (Leica DM microscope, 100 × objective, oil emulsion). **(A)** Breast tumor with *RRM1* gene deletion (FISH ratio < 0.8). **(B)** Breast tumor with *RRM2B* gene amplification (FISH ratio ≥ 2.0). Abbreviation: FISH, fluorescence *in situ* hybridization; *RRM1*, ribonucleotide reductase M1 subunit; *RRM2B*, ribonucleotide reductase M2B subunit.

The distribution of gene/centromere ratios are illustrated in Figure 2. *RRM1*/CEN-11 ratios were found in the range 0.13-3.35 (Figure 2A), and *RRM2B*/CEN-8 ratios in the range 0.43-7.33 (Figure 2B). Among the 251 patients, 38 patients (15.1%) had *RRM1* deletions (Figure 3A) and 2 (0.8%) had *RRM1* amplifications, whereas 8 patients (3.2%) had *RRM2B* deletions and 26 (10.4%) had *RRM2B* amplifications (Figure 3B). A total of 7 patients (2.8%) had an aberration of both genes (Table 1).

**Table 1:** ***RRM1 *** **and *RRM2B *** **status in 251 FISH assessable primary tumor samples from advanced breast cancer patients**

		***RRM1***		
***RRM2B***	Deletion	Normal	Amplification	Total
Deletion	3	5	0	8 (3.2%)
Normal	31	184	2	217 (86.4%)
Amplification	4	22	0	26 (10.4%)
	38 (15.1%)	211 (84.1%)	2 (0.8%)	251

*Abbreviations*: *FISH* fluorescence *in situ* hybridization, *RRM1* ribonucleotide reductase M1 subunit, *RRM2B* ribonucleotide reductase M2B subunit.

Patient and baseline characteristics according to *RRM1*, *RRM2B*, and 2R status were assessed. No association was found between *RRM1* and *RRM2B* status, and *RRM1* status was not associated with any other factors (Additional file 2: Table S2). *RRM2B* status was significantly associated with median age at randomization (P = 0.03), bone metastases (P = 0.03), *HER2* status (P = 0.01), prior chemotherapy for locally advanced or metastatic disease (P = 0.02) (Additional file 2: Table S2), and PAM50 subtype (P = 0.0004) (Table 2). All *RRM2B* aberrations except one were found in non-luminal A cancers (Table 2). The 2R status was significantly associated with median age at randomization (P = 0.02), stage of disease (P = 0.02), bone metastases (P = 0.02), *HER2* status (P = 0.04) (Additional file 2: Table S2), and PAM50 subtype (P = 0.009) (Table 2).

**Table 2:** **Association between *RRM1*, *RRM2B, *** **and 2R status and PAM50 intrinsic subtype**

**Characteristics**	***RRM1***					***RRM2B***					**2R**				
	**Normal**		**Aberrant**			**Normal**		**Aberrant**			**Normal**		**Aberrant**		
	**No.**	**(%)**	**No.**	**(%)**	**P**^**a**^	**No.**	**(%)**	**No.**	**(%)**	**P**^**a**^	**No.**	**(%)**	**No.**	**(%)**	**P**^**a**^
No. of patients	211		40			217		34			184		67		
**PAM50**^**b**^					0.38					0.0004					0.009
Luminal A	63	(29.9)	8	(20.0)		70	(32.3)	1	(2.9)		62	(33.7)	9	(13.4)	
Luminal B	74	(35.1)	15	(37.5)		75	(34.6)	14	(41.2)		62	(33.7)	27	(40.3)	
Basal-like	32	(15.2)	10	(25.0)		35	(16.1)	7	(20.6)		27	(14.7)	15	(22.4)	
HER2-enriched	37	(17.5)	7	(17.5)		33	(15.2)	11	(32.4)		29	(15.8)	15	(22.4)	
Unknown	5	(0.0)	0	(0.0)		4	(1.8)	1	(2.9)		4	(2.2)	1	(1.5)	

*Abbreviations*: *2R aberration RRM1* and/or *RRM2B* aberrant, *2R normal RRM1* and *RRM2B* both normal, *HER2* human epidermal growth factor receptor 2, *RRM1* ribonucleotide reductase M1 subunit, *RRM2B* ribonucleotide reductase M2B subunit.

^a^ Fishers exact test, unknown values excluded from tests.

^b^ PAM50: A 50-gene expression classifier that identifies the four major intrinsic subtypes of breast cancer known as the luminal A, luminal B, HER2-enriched and basal-like subtypes [26].

RR did not differ significantly according to status of *RRM1*, *RRM2B* or 2R (Table 3). However, a non-significant trend of a better overall RR was seen in patients with *RRM1* aberrations (32% *RRM1* normal; 50% *RRM1* aberrant, P = 0.08).

**Table 3:** **Best overall response**^**a **^**by *RRM1*, *RRM2B*, and 2R status**

**Response**	***RRM1***^***b***^				***RRM2B***^***c***^				**2R**^***d***^			
	**Normal**		**Aberrant**		**Normal**		**Aberrant**		**Normal**		**Aberrant**	
	**No.**	**(%)**	**No.**	**(%)**	**No.**	**(%)**	**No.**	**(%)**	**No.**	**(%)**	**No.**	**(%)**
**CR**	3	(2.3)	2	(7.1)	3	(2.2)	2	(9.1)	2	(1.8)	3	(6.7)
**PR**	39	(29.8)	12	(42.9)	45	(32.8)	6	(27.3)	36	(31.6)	15	(33.3)
Total responses	42	(32.0)	14	(50.0)	48	(35.0)	8	(36.4)	38	(33.3)	18	(40.0)
95% CI		(24.2 to 40.8)		(30.7 to 69.4)		(27.1 to 43.7)		(17.2 to 59.3)		(24.8 to 42.8)		(25.7 to 55.7)
**SD**	63	(48.1)	10	(35.7)	63	(46.0)	10	(45.4)	54	(47.4)	19	(42.2)
**PD**	18	(13.7)	1	(3.6)	15	(11.0)	4	(18.2)	14	(12.3)	5	(11.1)
Unknown	8	(6.1)	3	(10.7)	11	(8.0)	0	(0.0)	8	(7.0)	3	(6.7)
Total	131		28		137		22		114		45	

*Abbreviations*: *2R aberration RRM1* and/or *RRM2B* aberrant, *2R normal RRM1* and *RRM2B* both normal, *CI* confidence interval, *CR* complete response, *PD* progressive disease, *PR* partial response, *RRM1* ribonucleotide reductase M1 subunit, *RRM2B* ribonucleotide reductase M2B subunit, *SD* stable disease.

^a^Response rate was evaluated for patients with the presence of at least one measurable lesion (n = 159) according to RECIST (Response Evaluation Criteria in Solid Tumors), version 1.0.

^b^Total responses, Fishers exact test P = 0.08.

^c^Total responses, Fishers exact test P = 1.00.

^d^Total responses, Fishers exact test P = 0.46.

**Figure 4:**
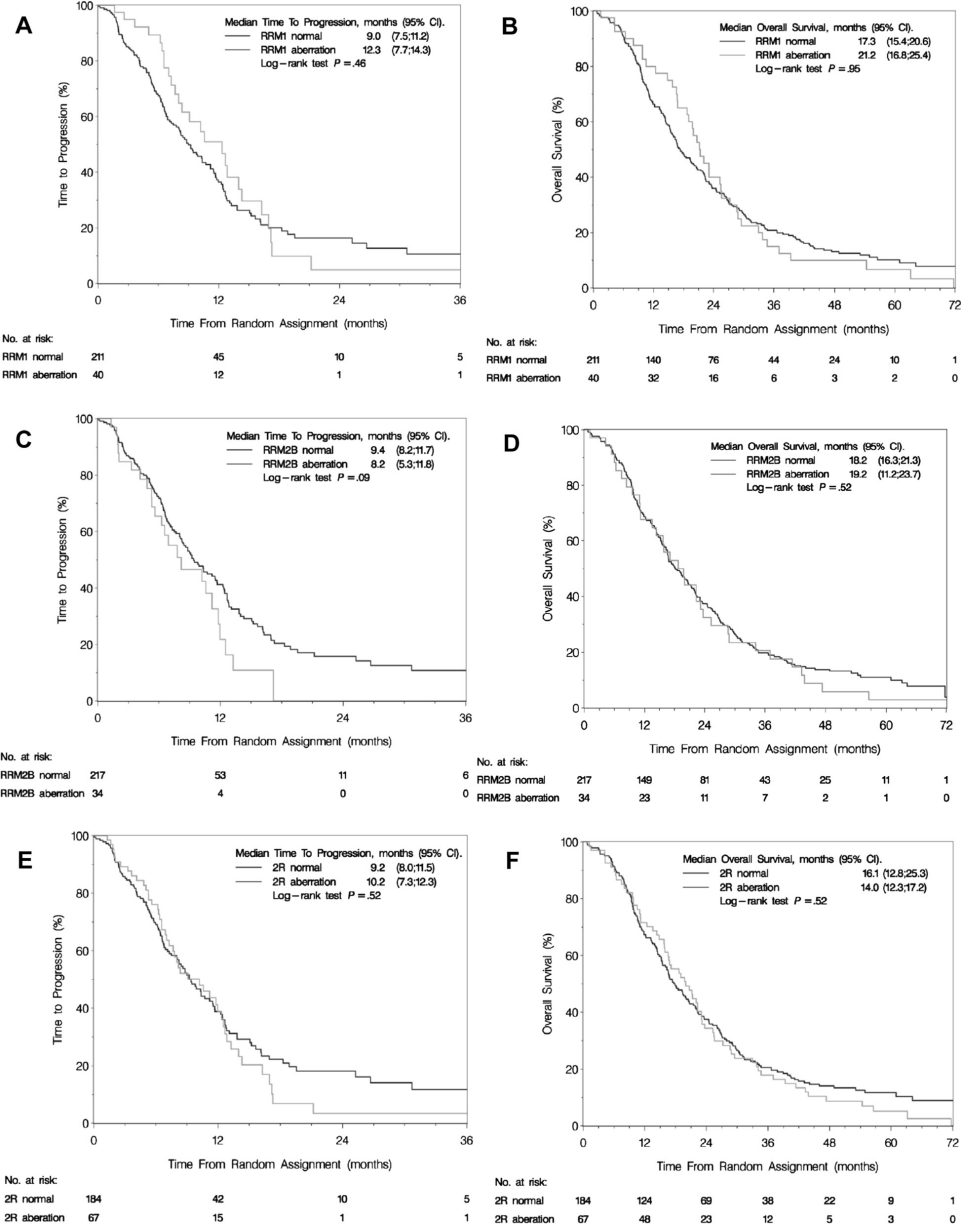
**Time to progression (164 events) and overall survival (228 events) of patients according to *RRM1*, *RRM2B*, and 2R status. (A)** TTP according to *RRM1* status. **(B)** OS according to *RRM1* status. **(C)** TTP according to *RRM2B* status. **(D)** OS according to *RRM2B* status. **(E)** TTP according to *RRM1* and *RRM2B* status combined. **(F)** OS according to *RRM1* and *RRM2B* status combined. Abbreviations: 2R aberration, *RRM1* and/or *RRM2B* aberrant; 2R normal, *RRM1* and *RRM2B* both normal; CI, confidence interval; OS, overall survival; *RRM1*, ribonucleotide reductase M1 subunit; *RRM2B*, ribonucleotide reductase M2B subunit; TTP, time to progression.

In Kaplan-Meier analyses, *RRM1*, *RRM2B* or 2R status were not significantly associated with TTP or OS (Figure 4). The Cox proportional hazards model for TTP and OS confirmed this result (Table 4). To meet the proportionality assumption of the Cox model, the OS model for *RRM1* was separated according to short time (0-1.5 years) and long time prognosis (1.5-7.4 years). Patients with *RRM1* aberrations tended to have a better OS in the time interval 0-1.5 years from randomization, though this difference was not statistically significant (hazard ratio (HR) = 0.59, 95% confidence interval (CI) = 0.34-1.03, P = 0.06). In contrary, *RRM1* aberrations were significantly associated with a poorer OS in the time interval 1.5-7.4 years (HR = 1.67, 95% CI = 1.06-2.62, P = 0.03). To test the independent value of *RRM1*, *RRM2B,* and 2R status against standard clinical and pathologic factors, multivariable Cox models were constructed. *RRM1* aberrations remained an independent prognostic factor with an impact on OS from 1.5-7.4 years from randomization (HR = 1.72, 95% CI = 1.05-2.79, P = 0.03) (Table 4).

**Table 4:** Cox univariate and multivariate analysis for time to progression and overall survival

**Risk factor**	**n**	**Time to progression**		**P**	**Overall survival**		**P**
		**HR**	**95% CI**		**HR**	**95% CI**	
**Univariate analysis**							
***RRM1***							
Normal	211	1.00	Referent		1.00	Referent	
Aberration	40	0.86	(0.57-1.29)	0.46			
Aberration 0–1.5 years*					0.59	(0.34-1.03)	0.06
Aberration 1.5-7.4 years*					1.67	(1.06-2.62)	0.03
***RRM2B***							
Normal	217	1.00	Referent		1.00	Referent	
Aberration	34	1.45	(0.94-2.25)	0.09	1.13	(0.78-1.64)	0.52
**2R**							
Normal	184	1.00	Referent		1.00	Referent	
Aberration	67	1.25	(0.92-1.69)	0.16			
Aberration 0–1.5 years*					0.85	(0.56-1.28)	0.44
Aberration 1.5-7.4 years*					1.44	(0.96-2.17)	0.08
**Multivariate analysis**^**a**^							
***RRM1***^***b***^							
Normal	211	1.00	Referent		1.00	Referent	
Aberration	40	0.66	(0.43-1.03)	0.07			
Aberration 0–1.5 years*					0.56	(0.32-0.99)	0.05
Aberration 1.5-7.4 years*					1.72	(1.05-2.79)	0.03
***RRM2B***^***c***^							
Normal	217	1.00	Referent		1.00	Referent	
Aberration	34	1.41	(0.88-2.23)	0.15	1.00	(0.68-1.47)	1.00
**2R**							
Normal	184	1.00	Referent		1.00	Referent	
Aberration	67	0.92	(0.64-1.33)	0.66			
Aberration 0–1.5 years*					0.80	(0.52-1.22)	0.30
Aberration 1.5-7.4 years*					1.36	(0.89-2.09)	0.16

*Abbreviations*: *2R aberration RRM1* and/or *RRM2B* aberrant, *2R normal RRM1* and *RRM2B* both normal, *CI* confidence interval, *HR* hazard ratio, *RRM1* ribonucleotide reductase M1 subunit, *RRM2B* ribonucleotide reductase M2B subunit.

^a^Models adjusted for the effects of PAM50 status, visceral disease, stage of disease, number of metastatic sites, and performance status. Models stratified for previous chemotherapy (none, n = 70; adjuvant, n = 86; locally advanced or metastatic, n = 95).

^b^Model further adjusted for *RRM2B*.

^c^Model further adjusted for *RRM1*.

*Time-dependant variable. If nothing is mentioned the maximum follow-up is 7.4 years after randomization for OS and 6.0 years after randomization for TTP.

Treatment effect of GD versus D was comparable to the effect in the original study with regard to TTP (adjusted HR 0.56; 95% CI, 0.40-0.79, P = 0.001) and OS (HR 0.84; 95% CI, 0.64-1.12, P = 0.23) [19]. The subgroup analyses showed no treatment effect heterogeneity on the TTP and OS endpoints according to *RRM1* (TTP, P_interaction_ = 0.35; OS, P_interaction_ = 0.50), *RRM2B* (TTP, P_interaction_ = 0.60; OS, P_interaction_ = 0.63), or 2R (TTP, P_interaction_ = 0.24; OS, P_interaction_ = 0.82).

The explorative definition of amplification and deletion cut-points resulted in a higher proportion of *RRM1* and *RRM2B* aberrations but did not alter the results overall (data not shown).

For assay validation 60 normal breast samples were analyzed for *RRM1* and *RRM2B* gene copy number variation. Neither amplifications nor deletions were found. For each sample 60+ events (1 event = 1 red/gene signal) was evaluated, with an average of 37.25 and 36.78 nuclei for *RRM1* and *RRM2B,* respectively. An average of 1.62 red signals representing the *RRM1* genes and 1.55 green signals representing the centromeric sequence were counted. The *RRM1*/CEN-11 ratio varied from 0.92-1.20. An average of 1.64 red signals representing the *RRM2B* genes and 1.55 green signals representing the centromeric sequence were counted. The *RRM2B*/CEN-8 ratio varied from 0.94-1.15. The ratios were distributed normally with a standard deviation of 0.05 (data not shown).

## Discussion

This study demonstrated the existence of both deletions and amplifications of *RRM1* and *RRM2B* in primary breast tumor samples, to our knowledge unprecedented in human tumor tissue but in agreement with the frequently observed somatic changes in the genome of breast cancer cell lines [27-29]. *RRM1* aberrations were demonstrated to be a time-dependent prognostic factor with an impact on OS, and *RRM2B* aberrations were significantly associated with *HER2* status and PAM50 subtype. In this study we found no heterogeneity of treatment effects according to *RRM1* or *RRM2B* copy number status in terms of TTP or OS. Combining *RRM1* and *RRM2B* aberrations did not add further information.

We found deletions to be the most frequent gene copy number variation in relation to *RRM1.* The *RRM1* gene resides at 11p15.5, a chromosomal region frequently associated with allelic loss in cancer [30,31] and agrees well with the *RRM1* deletions frequently observed in this study. Low level of RRM1 in early-stage cancer patients treated with surgery only has been associated with reduced survival [32-35], whereas low RRM1 expression in gemcitabine-treated advanced cancer patients has been associated with improved survival [6,9]. The prognostic impact of RRM1 expression has not been confirmed in a randomized trial. A prospective randomized phase III trial by Reynolds et al (2009) [11] failed to demonstrate substantial difference in survival according to RRM1 levels in lung cancer patients treated with gemcitabine and carboplatin or gemcitabine monotherapy. The retrospective biomarker sub-study of another randomized phase III trial did not find a prognostic impact of RRM1 protein expression in a subgroup of lung cancer patients randomly assigned to cisplatin-based chemotherapy including gemcitabine [13]. In the present study *RRM1* was demonstrated to be a time-dependent prognostic factor with an impact on OS 1.5-7.4 years from randomization (HR = 1.72, P = 0.03). *RRM1* aberrations were significantly associated with a decreased OS of the patients during this time interval. Though not statistically significant an inverse trend in OS was seen for patients with *RRM1* aberrations 0-1.5 years from randomization. Differences in methodologies, chemotherapy regimens, and cancer type do compromise the comparability of studies, and the prognostic significance of RRM1 in advanced breast cancer remains to be further elucidated.

Amplifications were the most common gene copy number variation in relation to *RRM2B* observed in this study. This is in agreement with the location of *RRM2B* at 8q22.3 which is a chromosomal region commonly characterized by DNA copy number gains [27,36]. Several studies have pointed out that the gain of 8q is a recurrent event in sporadic breast cancer with poor prognosis [36,37]. An association between RRM2B expression and increased survival has been noticed in lung and colon cancer [38-40], whereas an association with poor prognosis has been observed in esophageal cancer patients [41]. *RRM2B* aberrations in this study did not show any prognostic impact, in agreement with one clinical study by Uramoto et al. (2006) [42] that found no support for an association between RRM2B protein expression and survival outcome in stage I-III lung cancer patients. Hence, there was a non-significant trend that patients with *RRM2B* amplifications or deletions had disease progression earlier than patients with normal *RRM2B* status.

Moreover, the predictive value of RRM1 and RRM2B in relation to gemcitabine-containing versus non-gemcitabine-containing therapy has not received much attention, as only few and very small retrospective studies have compared the effect of gemcitabine with a control/gemcitabine naïve group [32]. More recently, an international phase III trial that utilized levels of RRM1 to assign lung cancer patients to a gemcitabine-containing regimen if RRM1 levels were low and a docetaxel-containing regimen if RRM1 levels were high versus a control arm of unselected chemotherapy consisting of carboplatin plus gemcitabine, did not demonstrate a survival or response rate benefit from individualizing therapy [43]. Our data did not show a significant interaction between *RRM1* and/or *RRM2B* copy number status and outcome from adding gemcitabine to docetaxel in terms of TTP or OS. An interesting aspect in the context of the gemcitabine-taxane chemotherapy combination could be the coexpression of RRM1 and BRCA1 previously observed in metastatic breast cancer specimens [44]. Where RRM1 low/negative expression has been connected to gemcitabine sensitivity, decreased BRCA1 expression may enhance the resistance to anti-microtubule agents [45]. Hence, the clinical specimens investigated in this study may appear suboptimal to provide results indicating an interaction between RRM1 and the clinical outcome of gemcitabine.

*RRM2B* status was significantly associated with *HER2* status. Almost 30% of the tumors with a *RRM2B* aberration also had *HER2* amplification. Several previous studies have shown a nonrandom accumulation of amplifications of different genomic regions in certain breast cancers that are considered to show an ‘amplifier’ phenotype [20,27,36,46,47]. Aberrations at the 8q22 locus are probably also part of a spectrum of breast carcinomas with high genomic instability and frequent amplifications. In addition, this is in agreement with *RRM2B* status being significantly associated with gene expression classified intrinsic subtype, as all but one aberration were seen to accumulate in the more proliferating non-luminal A subtypes [20,21]. This is, however, in contrast to *RRM1* copy number variation, which was not significantly different between the subtypes of breast cancer. A previous study by Kim et al. (2011) [48] where the expression of RRM1 protein in breast cancer samples was evaluated by immunohistochemical classification reached the same conclusion. The relatively frequent aberrations observed in this study imply that *RRM1* and *RRM2B* are capable of contributing to breast cancer development. The aberrations were not associated suggesting that the aberrations characterize two distinct geno-subtypes with different genetic pathways in the evolution of invasive breast cancer.

The strengths of the study include data from more than 74% of the participants from a randomized trial, prospectively defined hypotheses and analysis plan, long term follow-up, biomarker analysis blinded from clinical outcome, and outcome analysis by an independent statistical core. However, the statistical power was limited due to the small population size, especially under-powering the results of the subgroup analysis. Furthermore, a potential limitation concerns the fact that the predictive value of gene aberrations is evaluated upon the primary tumor profile, although the molecular portrait could have changed pronouncedly between primary and metastatic disease [49,50]. Moreover, one has to consider the limitations of conclusions based on an evaluation at the genomic level only. This study focused on gene copy number changes without correlation with gene expression. Also, point mutations, not detectable by the FISH assay utilized in this study, could be of significant value [51,52], as well as several factors but genetic alterations could influence the expression and activity of the enzyme proteins, such as posttranscriptional and posttranslational regulation.

## Conclusions

In summary, this study revealed the occurrence of *RRM1* and *RRM2B* aberrations in primary breast tumor specimens. We found no support of a differential outcome according to these aberrations in advanced breast cancer patients randomized to the combination of gemcitabine and docetaxel as compared to docetaxel alone.

## Abbreviations

2R aberration: *RRM1* and/or *RRM2B* aberrant; 2R normal: *RRM1* and *RRM2B* both normal; BAC: Bacterial artificial chromosome; CEN-8: Centromeric probe for chromosome 8; CEN-11: Centromeric probe for chromosome 11; CI: Confidence interval; D: Docetaxel; DBCG: Danish breast cancer cooperative group; ECOG: Eastern cooperative oncology group; FISH: Fluorescence *in situ* hybridization; FFPE: Formalin-fixed, paraffin-embedded; G: Gemcitabine; HER2: Human epidermal growth factor receptor 2; HR: Hazed ratio; OS: Overall survival; PNA: Peptide nucleic acid; RR: Response rate; RNR: Ribonucleotide reductase; RRM1: Ribonucleotide reductase M1 subunit; RRM2: Ribonucleotide reductase M2 subunit; RRM2B: Ribonucleotide reductase M2B subunit; TMA: Tissue micro array; TOP2A: DNA topoisomerase II-alpha; TTP: Time to progression.

## Competing interests

The authors declare that they have no competing interests.

## Authors’ contributions

KVN, BE, EB, and CLTJ made contributions to conception and design of the study. DLN and BE provided access to clinical data on study material, and EB and CLTJ collected the tissue samples and constructed the TMAs. CLTJ performed FISH analysis and evaluation of samples. KDB and CLTJ performed the statistical analysis. All authors contributed to analysis and interpretation of data. CLTJ and KVN drafted the manuscript, and all authors contributed to the manuscript preparation and in revising the manuscript critically. All authors read and approved the final manuscript.

## Pre-publication history

The pre-publication history for this paper can be accessed here:

http://www.biomedcentral.com/1471-2407/13/541/prepub

## Supplementary Material

Additional file 1: Table S1 Patient demographics, disease characteristics, and prior therapy for excluded versus included patients. Click here for file

Additional file 2: Table S2 Association between *RRM1, RRM2B*, and 2R status and patient demographics, disease characteristics, and prior therapy. Click here for file
