# Supplementary material for: Gene aberrations of RRM1 and RRM2B and outcome of advanced breast cancer after treatment with docetaxel with or without gemcitabine
Source: BMC Cancer. 2013 Nov 12;13:541. doi: 10.1186/1471-2407-13-541 (PMC3840598; doi:10.1186/1471-2407-13-541)
Supplement: Additional file 1: Table S1 — Patient demographics, disease characteristics, and prior therapy for excluded versus included patients. [file 1471-2407-13-541-S1.docx]

| **Additional file 1: Table S1.**  Patient demographics, disease characteristics, and prior therapy for excluded versus included patients | | | | | | |  |
| --- | --- | --- | --- | --- | --- | --- | --- |
|  | **Included** | |  | **Excluded** | |  |  |
| Characteristics | No. | (%) |  | No. | (%) |  | P^a^ |
| No. of patients | 251 |  |  | 86 |  |  |  |
|  |  |  |  |  |  |  |  |
| **Regimen** |  |  |  |  |  |  | 0.13 |
| Gemcitabine and docetaxel | 133 | (53.0) |  | 37 | (43.0) |  |  |
| Docetaxel | 118 | (47.0) |  | 49 | (57.0) |  |  |
|  |  |  |  |  |  |  |  |
| **Median age at randomization** |  |  |  |  |  |  | 0.74^b^ |
| Years | 59 | |  | 57 | |  |  |
| Range | 30-74 | |  | 36-75 | |  |  |
|  |  |  |  |  |  |  |  |
| **ECOG performance status** |  |  |  |  |  |  | 0.70 |
| 0-1 | 211 | (84.1) |  | 77 | (89.5) |  |  |
| 2 | 31 | (12.4) |  | 9^c^ | (10.5) |  |  |
| Unknown | 9 | (3.6) |  | 0 | (0.0) |  |  |
|  |  |  |  |  |  |  |  |
| **Stage of disease** |  |  |  |  |  |  | 0.21 |
| Locally advanced | 22 | (8.8) |  | 12 | (14.0) |  |  |
| Metastatic | 229 | (91.2) |  | 74 | (86.0) |  |  |
|  |  |  |  |  |  |  |  |
| **No. of metastatic sites** |  |  |  |  |  |  | 0.85 |
| 1 | 73 | (29.1) |  | 23 | (26.7) |  |  |
| 2 | 90 | (35.9) |  | 30 | (34.9) |  |  |
| ≥3 | 88 | (35.1) |  | 33 | (38.4) |  |  |
|  |  |  |  |  |  |  |  |
| **Type of metastatic site** |  |  |  |  |  |  |  |
| Visceral | 146 | (58.2) |  | 47 | (54.7) |  | 0.61 |
| Lung | 74 | (29.5) |  | 29 | (33.7) |  | 0.42 |
| Liver | 96 | (38.2) |  | 30 | (34.9) |  | 0.61 |
| Non-visceral | 105 | (41.8) |  | 39 | (45.3) |  |  |
| Bone | 163 | (64.9) |  | 53 | (61.6) |  | 0.60 |
|  |  |  |  |  |  |  |  |
| **Hormone receptor status** |  |  |  |  |  |  | 0.78 |
| Positive | 179 | (71.3) |  | 60 | (69.8) |  |  |
| Negative | 69 | (27.5) |  | 21 | (24.4) |  |  |
| Unknown | 3 | (1.2) |  | 5 | (5.8) |  |  |
|  |  |  |  |  |  |  |  |
| ***HER2* status**^d^ |  |  |  |  |  |  | 0.25 |
| Normal/deletion | 212 | (84.5) |  | 20 | (23.3) |  |  |
| Amplification | 35 | (13.9) |  | 6 | (7.0) |  |  |
| Unknown | 4 | (1.6) |  | 60 | (69.8) |  |  |
|  |  |  |  |  |  |  |  |
| **Prior chemotherapy** |  |  |  |  |  |  |  |
| Total | 181 | (72.1) |  | 52 | (60.5) |  | 0.06 |
| (Neo)adjuvant | 124 | (49.4) |  | 20 | (23.3) |  | <0.0001 |
| Anthracycline | 72 | (28.7) |  | 10 | (11.6) |  |  |
| Non-anthracycline | 52 | (20.7) |  | 10 | (11.6) |  |  |
| Locally advanced/metastatic | 95 | (37.8) |  | 38 | (44.2) |  | 0.31 |
| Anthracycline | 81 | (32.3 |  | 35 | (40.7) |  |  |
| Non-anthracycline | 14 | (5.5) |  | 3 | (3.5) |  |  |
|  |  |  |  |  |  |  |  |
| **Hormonal therapy** |  |  |  |  |  |  |  |
| Total | 161 | (64.1) |  | 53 | (61.6) |  | 0.70 |
| Adjuvant | 115 | (45.8) |  | 23 | (26.7) |  | 0.003 |
| Locally advanced/metastatic | 110 | (43.8) |  | 47 | (54.7) |  | 0.10 |
|  |  |  |  |  |  |  |  |
| **Radiotherapy** | 154 | (61.4) |  | 25 | (29.1) |  | <0.0001 |
|  |  |  |  |  |  |  |  |
| **Disease-free interval, months**^e^ | |  |  |  |  |  |  |
| Median | 30 | |  | 32 | |  | 0.74^b^ |
| Range | 0-250 | |  | 0-231 | |  |  |
| Abbreviations: ECOG, Eastern Cooperative Oncology Group; *HER2*, human epidermal growth factor | | | | | | | |
| receptor 2. |  |  |  |  | |  |  |
|  |  |  |  |  | |  |  |
| ^a^ Fishers exact test, unknown values excluded from tests. | | | |  |  |  |  |
| ^b^ Wilcoxon test.  ^c^ Including one patient ECOG performance 3.  ^d^ Retrospective analysis, primary tumor only.  ^e^ Time interval from diagnosis of primary cancer to recurrence. |  |  |  |  |  |  |  |
|  | | | | |  |  |  |
