# Supplementary material for: Gene aberrations of RRM1 and RRM2B and outcome of advanced breast cancer after treatment with docetaxel with or without gemcitabine
Source: BMC Cancer. 2013 Nov 12;13:541. doi: 10.1186/1471-2407-13-541 (PMC3840598; doi:10.1186/1471-2407-13-541)
Supplement: Additional file 2: Table S2 — Association between RRM1, RRM2B, and 2R status and patient demographics, disease characteristics, and prior therapy. [file 1471-2407-13-541-S2.docx]

| **Additional file 2: Table S2.**  Association between *RRM1*, *RRM2B* and 2R status and patient demographics, disease characteristics, and prior therapy | | | | | | | | | | | | | | | | | |
| --- | --- | --- | --- | --- | --- | --- | --- | --- | --- | --- | --- | --- | --- | --- | --- | --- | --- |
|  | ***RRM1*** | | | | |  | ***RRM2B*** | | | | |  | **2R** | | | | |
|  | Normal | | Aberrant | |  |  | Normal | | Aberrant | |  |  | Normal | | Aberrant | |  |
| Characteristics | No. | (%) | No. | (%) | P^a^ |  | No. | (%) | No. | (%) | P^a^ |  | No. | (%) | No. | (%) | P^a^ |
| No. of patients | 211 |  | 40 |  |  |  | 217 |  | 34 |  |  |  | 184 |  | 67 |  |  |
|  |  |  |  |  |  |  |  |  |  |  |  |  |  |  |  |  |  |
| ***RRM2B*** |  |  |  |  | 0.45 |  |  |  |  |  |  |  |  |  |  |  |  |
| Normal | 184 | (87.2) | 33 | (82.5) |  |  |  |  |  |  |  |  |  |  |  |  |  |
| Aberrant | 27 | (12.8) | 7 | (17.5) |  |  |  |  |  |  |  |  |  |  |  |  |  |
|  |  |  |  |  |  |  |  |  |  |  |  |  |  |  |  |  |  |
| **Regimen** |  |  |  |  | 0.73 |  |  |  |  |  | 0.36 |  |  |  |  |  | 1.00 |
| Gemcitabine and docetaxel | 113 | (53.6) | 20 | (50.0) |  |  | 112 | (51.6) | 21 | (61.8) |  |  | 97 | (52.7) | 36 | (53.7) |  |
| Docetaxel | 98 | (46.4) | 20 | (50.0) |  |  | 105 | (48.4) | 13 | (38.2) |  |  | 87 | (47.3) | 31 | (46.3) |  |
|  |  |  |  |  |  |  |  |  |  |  |  |  |  |  |  |  |  |
| **Median age at randomization** |  |  |  |  | 0.10^b^ |  |  |  |  |  | 0.03^b^ |  |  |  |  |  | 0.02^b^ |
| Years | 58 | | 62 | |  |  | 58 | | 61 | |  |  | 58 | | 61 | |  |
| Range | 30-74 | | 41-71 | |  |  | 30-74 | | 38-73 | |  |  | 30-74 | | 38-73 | |  |
|  |  |  |  |  |  |  |  |  |  |  |  |  |  |  |  |  |  |
| **ECOG performance status** |  |  |  |  | 0.43 |  |  |  |  |  | 0.59 |  |  |  |  |  | 1.00 |
| 0-1 | 176 | (83.4) | 35 | (87.5) |  |  | 183 | (84.3) | 28 | (82.4) |  |  | 154 | (83.7) | 57 | (85.1) |  |
| 2 | 28 | (13.3) | 3 | (7.5) |  |  | 26 | (12.0) | 5 | (14.7) |  |  | 23 | (12.5) | 8 | (11.9) |  |
| Unknown | 7 | (3.3) | 2 | (5.0) |  |  | 8 | (3.7) | 1 | (2.9) |  |  | 7 | (3.8) | 2 | (3.0) |  |
|  |  |  |  |  |  |  |  |  |  |  |  |  |  |  |  |  |  |
| **Stage of disease** |  |  |  |  | 0.06 |  |  |  |  |  | 0.19 |  |  |  |  |  | 0.02 |
| Locally advanced | 15 | (7.1) | 7 | (17.7) |  |  | 17 | (7.8) | 5 | (14.7) |  |  | 11 | (6.0) | 11 | (16.4) |  |
| Metastatic | 196 | (92.9) | 33 | (82.5) |  |  | 200 | (92.2) | 29 | (85.3) |  |  | 173 | 94.0) | 56 | (83.6) |  |
|  |  |  |  |  |  |  |  |  |  |  |  |  |  |  |  |  |  |
| **No. of metastatic sites** |  |  |  |  | 0.93 |  |  |  |  |  | 0.36 |  |  |  |  |  | 0.85 |
| 1 | 61 | (28.9) | 12 | (30.0) |  |  | 65 | (30.0) | 8 | (23.5) |  |  | 54 | (29.3) | 19 | (28.4) |  |
| 2 | 75 | (35.5) | 15 | (37.5) |  |  | 74 | (34.1) | 16 | (47.1) |  |  | 64 | (34.8) | 26 | (38.8) |  |
| ≥3 | 75 | (35.5) | 13 | (32.5) |  |  | 78 | (35.9) | 10 | (29.4) |  |  | 66 | (35.9) | 22 | (32.8) |  |
|  |  |  |  |  |  |  |  |  |  |  |  |  |  |  |  |  |  |
| **Type of metastatic site** |  |  |  |  |  |  |  |  |  |  |  |  |  |  |  |  |  |
| Visceral | 122 | (57.8) | 24 | (60.0) | 0.86 |  | 127 | (58.5) | 19 | (55.9) | 0.85 |  | 107 | (58.2) | 39 | (58.2) | 1.00 |
| Lung | 60 | (28.4) | 14 | (35.0) | 0.45 |  | 60 | (27.6) | 14 | (41.2) | 0.10 |  | 72 | (39.1) | 34 | (50.7) | 0.11 |
| Liver | 84 | (39.8) | 12 | (30.0) | 0.37 |  | 87 | (40.1) | 9 | (26.5) | 0.18 |  | 76 | (41.3) | 20 | (29.9) | 0.14 |
| Non-visceral | 89 | (42.2) | 16 | (40.0) |  |  | 90 | (41.5) | 15 | (44.1) |  |  | 77 | (41.8) | 28 | (41.8) |  |
| Bone | 140 | (66.4) | 23 | (57.5) | 0.28 |  | 147 | (67.7) | 16 | (47.1) | 0.03 |  | 128 | (69.6) | 35 | (52.2) | 0.02 |
|  |  |  |  |  |  |  |  |  |  |  |  |  |  |  |  |  |  |
| **Hormone receptor status** |  |  |  |  | 0.18 |  |  |  |  |  | 0.14 |  |  |  |  |  | 0.15 |
| Positive | 154 | (73.0) | 25 | (62.5) |  |  | 159 | (73.3) | 20 | (58.8) |  |  | 136 | (73.9) | 43 | (64.2) |  |
| Negative | 54 | (25.6) | 15 | (37.5) |  |  | 56 | (25.8) | 13 | (38.2) |  |  | 46 | (25.0) | 23 | (34.3) |  |
| Unknown | 3 | (1.4) | 0 | (0.0) |  |  | 2 | (0.9) | 1 | (2.9) |  |  | 2 | (1.1) | 1 | (1.5) |  |
|  |  |  |  |  |  |  |  |  |  |  |  |  |  |  |  |  |  |
| ***HER2* status^c^** |  |  |  |  | 0.32 |  |  |  |  |  | 0.01 |  |  |  |  |  | 0.04 |
| Normal/deletion | 180 | (85.3) | 32 | (80.0) |  |  | 188 | (86.6) | 24 | (70.6) |  |  | 160 | (87.0) | 52 | (77.6) |  |
| Amplification | 27 | (12.8) | 8 | (20.0) |  |  | 25 | (11.5) | 10 | (29.4) |  |  | 20 | (10.9) | 15 | (22.4) |  |
| Unknown | 4 | (1.9) | 0 | (0.0) |  |  | 4 | (1.8) | 0 | (0.0) |  |  | 4 | (2.2) | 0 | (0.0) |  |
|  |  |  |  |  |  |  |  |  |  |  |  |  |  |  |  |  |  |
| **Prior chemotherapy** |  |  |  |  |  |  |  |  |  |  |  |  |  |  |  |  |  |
| Total | 156 | (73.9) | 25 | (62.5) | 0.18 |  | 153 | (70.5) | 28 | (82.4) | 0.22 |  | 134 | (72.8) | 47 | (70.1) | 0.75 |
| (Neo)adjuvant | 107 | (50.7) | 17 | (42.5) | 0.39 |  | 106 | (48.8) | 18 | (52.9) | 0.71 |  | 94 | (51.1) | 30 | (44.8) | 0.40 |
| Anthracycline | 64 | (30.3) | 8 | (20.0) |  |  | 65 | (30.0) | 7 | (20.6) |  |  | 58 | (31.5) | 14 | (20.9) |  |
| Non-anthracycline | 43 | (20.4) | 9 | (22.5) |  |  | 41 | (18.9) | 11 | (32.4) |  |  |  |  |  |  |  |
| Locally advanced/metastatic | 78 | (37.0) | 17 | (42.5) | 0.59 |  | 76 | (35.0) | 19 | (55.9) | 0.02 |  | 64 | (34.8) | 31 | (46.3) | 0.11 |
| Anthracycline | 69 | (32.7) | 12 | (30.0) |  |  | 66 | (30.4) | 15 | (44.1) |  |  | 56 | (30.4) | 25 | (37.3) |  |
| Non-anthracycline | 9 | (4.3) | 5 | (12.5) |  |  | 10 | (4.6) | 4 | (11.8) |  |  | 8 | (4.3) | 6 | (9.0) |  |
|  |  |  |  |  |  |  |  |  |  |  |  |  |  |  |  |  |  |
| **Hormonal therapy** |  |  |  |  |  |  |  |  |  |  |  |  |  |  |  |  |  |
| Total | 137 | (64.9) | 24 | (60.0) | 0.59 |  | 143 | (65.9) | 18 | (52.9) | 0.18 |  | 121 | (65.8) | 40 | (59.7) | 0.56 |
| Adjuvant | 98 | (46.4) | 17 | (42.5) | 0.61 |  | 102 | (47.0) | 13 | (38.2) | 0.70 |  | 86 | (46.7) | 29 | (43.3) | 0.88 |
| Locally advanced/metastatic | 93 | (44.1) | 17 | (42.5) | 1.00 |  | 98 | (45.2) | 12 | (35.3) | 0.35 |  | 83 | (45.1) | 27 | (40.3) | 0.57 |
|  |  |  |  |  |  |  |  |  |  |  |  |  |  |  |  |  |  |
| **Radiotherapy** | 132 | (62.6) | 22 | (55.0) | 0.38 |  | 134 | (61.8) | 20 | (58.8) | 0.85 |  | 116 | (63.0) | 38 | (56.7) | 0.38 |
|  |  |  |  |  |  |  |  |  |  |  |  |  |  |  |  |  |  |
| **Disease-free interval, months** |  |  |  |  | 0.33^b^ |  |  |  |  |  | 0.56^b^ |  |  |  |  |  | 0.81^b^ |
| Median | 31 | | 27 | |  |  | 30 | | 30 | |  |  | 30 | | 29 | |  |
| Range | 0-250 | | 0-122 | |  |  | 0-250 | | 0-165 | |  |  | 0-250 | | 0-165 | |  |
|  |  |  |  |  |  |  |  |  |  |  |  |  |  |  |  |  |  |
| Abbreviations: ECOG, Eastern Cooperative Oncology Group; *HER2*, human epidermal growth factor receptor 2; *RRM1*, ribonucleotide reductase M1 subunit; *RRM2B*, ribonucleotide reductase M2B subunit; 2R, *RRM1* and *RRM2B* both normal v one or both aberrant; *RRM1*, ribonucleotide reductase M1 subunit; *RRM2B*, ribonucleotide reductase M2B subunit. | | | | | | | | | | | | | | | | |  |
| ^a^ Fishers exact test, unknown values excluded from tests. | | | | |  |  |  |  |  |  |  |  |  |  |  |  |  |
| ^b^ Wilcoxon test.  ^c^ Retrospective analysis, primary tumor only.  ^d^ Time interval from diagnosis of primary cancer to recurrence. |  |  |  |  |  |  |  |  |  |  |  |  |  |  |  |  |  |
